# Actual Therapeutic Indication of an Old Drug: Urea for Treatment of Severely Symptomatic and Mild Chronic Hyponatremia Related to SIADH

**DOI:** 10.3390/jcm3031043

**Published:** 2014-09-18

**Authors:** Guy Decaux, Fabrice Gankam Kengne, Bruno Couturier, Frédéric Vandergheynst, Wim Musch, Alain Soupart

**Affiliations:** 1 Departments of Internal Medicine, Erasmus University Hospital, Route de Lennik, 808, Brussels B-1070, Belgium; E-Mails: bruno.couturier@erasme.ulb.ac.be (B.C.); frederic.vandergheynst@erasme.ulb.ac.be (F.V.); alain.soupart@entitejolimontoise.be (A.S.); 2 Departments of Nephrology, Erasmus University Hospital, Brussels B-1070, Belgium; E-Mail: fabrice.gankam@erasme.ulb.ac.be; 3 Department of Internal Medicine, Bracops Hospital, Brussels B-1070, Belgium; E-Mail: wim.musch@numericable.be; 4 Department of Internal Medicine, Jolimont/Tubize Hospital, Tubize 1480, Belgium

**Keywords:** hyponatremia, urea, V2 antagonist, ODS, SIADH, high ceiling diuretics

## Abstract

Oral urea has been used in the past to treat various diseases like gastric ulcers, liver metastases, sickle cell disease, heart failure, brain oedema, glaucoma, Meniere disease, *etc*. We have demonstrated for years, the efficacy of urea to treat euvolemic (SIADH) or hypervolemic hyponatremia. We briefly describe the indications of urea use in symptomatic and paucisymptomatic hyponatremic patients. Urea is a non-toxic, cheap product, and protects against osmotic demyelinating syndrome (ODS) in experimental studies. Prospective studies showing the benefit to treat mild chronic hyponatremia due to SIADH and comparing water restriction, urea, high ceiling diuretics, and antivasopressin antagonist antagonist should be done.

## 1. Introduction

Urea is the main nitrogen-containing substance in the urine of man. It is highly soluble in water and practically non-toxic (LD_50_ is 15 g/kg for rat). It represents about half of our daily osmotic load eliminated in the urine. It is synthesized in the liver (“urea cycle”) and corresponds to the catabolism of amino-acids in excess of our needs (coming from food) and which are not used by the organism (mainly protein synthesis) [1].

Urine therapy is an old-age practice, still use by millions of people over the world with the belief that it has a positive impact on health. Oral urea has been used to treat gastritis and gastric ulcers (even during bleeding) in the past, and, for years [2,3], liver metastases [4], prophylactic treatment of sickle cell disease [5], hematuria related to drepanocytosis [6], and heart failure [7,8].

It has been mainly used to treat brain oedema [9,10,11], glaucoma [10], and Meniere disease [12]. Urea has also been proposed to treat euvolemic [13,14,15] or hypervolemic hyponatremia [16,17,18]. It can be used orally [13] or intravenously [19]. We will briefly discuss the use of urea in the acute or chronic treatment of euvolemic hyponatremia, mainly orally.

## 2. Use of Urea in Severely Symptomatic Hyponatremia

Urea has been used orally or intravenously over time as an osmotic diuretic drug and as an agent to reduce intracranial and intraocular pressure [10].

As opposed to mannitol, urea enters intracellular spaces rapidly (in less than one hour) throughout the body, decreasing the immediate risk of sudden cardiac decompensation due to rapid intravascular volume expansion and does not induce a transient decrease in serum sodium (SNa) as observed with mannitol (translocation hyponatremia) [9].

However, because urea penetrates into the central nervous system (CNS) only about one-tenth as quickly as into muscle, a significant intravascular to CNS urea gradient occurs (during 4 to 10 h) [20]. Decreases in brain water content and intracranial pressure during urea administration have been measured experimentally and/or clinically [10].

High doses of urea can be given on a long-term basis without renal toxicity, which is not the case for mannitol.

In individuals with previously normal baseline renal function, the mean total dose of mannitol that precipitated acute renal failure was 626 ± 270 g over two to five days [21] (which represents about 209 ± 90 g urea on an equimolar basis). With persistent syndrome of inappropriate secretion of antidiuretic hormone (SIADH) (which is frequent with different brain diseases) urea can also be used in a long-term without toxicity [22,23].

**Figure 1:**
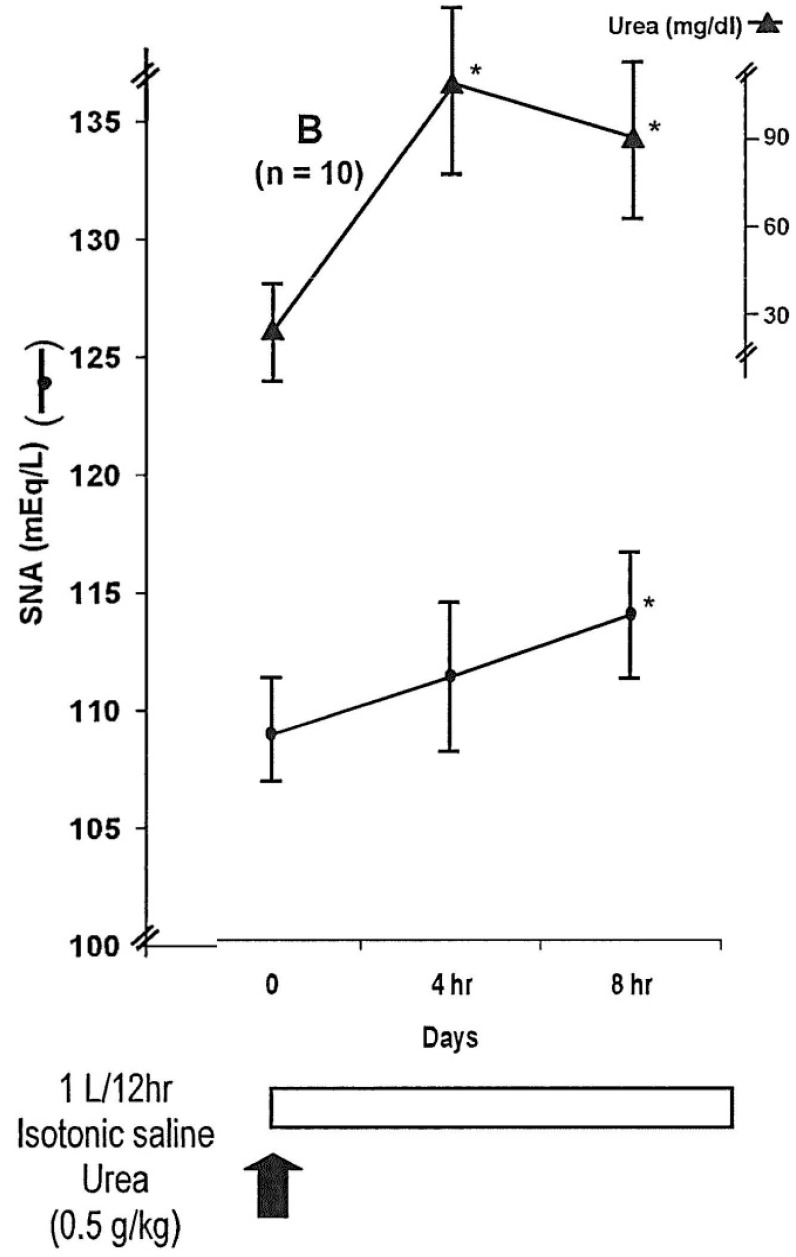
Evolution of SNa and urea (mean ± SD) each four hours in 10 patients with severe hyponatremia (<115 mEq/L) related to SIADH of various origins (adapted from reference [25] with permission from the authors, Decaux *et al.*).

We are using urea, mainly orally (by gastric tube if needed). Oral urea is less expensive than the intravenous route [24]. Figure 1 shows the evolution of SNa (mean ± SD) every four hours in 10 patients with severe hyponatremia (<115 mmol/L) due to SIADH and neurological symptoms (somnolence, confusion, *etc*.) treated with 1 L isotonic saline over 12 h and urea (0.5 g/kg) administered by gastric tube over 5–10 min. In these patients, mean SNa increased by 7 mmol/L in eight hours. All the patients made a rapid neurological recovery [25]. At the present time, severe symptomatic hyponatremia, particularly if epileptic seizures are present, should be treated with hypertonic saline (consensus conference) [26]. Hypertonic saline will theoretically increase SNa more rapidly than urea. We can expect that acute administration of urea at 0.5 g/kg body weight (b.w.) intravenously over one hour, or orally, will rapidly (one hour) increase serum osmolality by 15 mOsm/kg/H_2_O during a few hours (renal elimination) [25] and will decrease intracranial pressure during a few hours [10]. We advise, even in acute hyponatremia (<48 h), a daily increase of SNa of less than 10 mmol/L/24 h.

## 3. Treatment of Chronic Hyponatremia Related to SIADH by Urea

**Figure 2:**
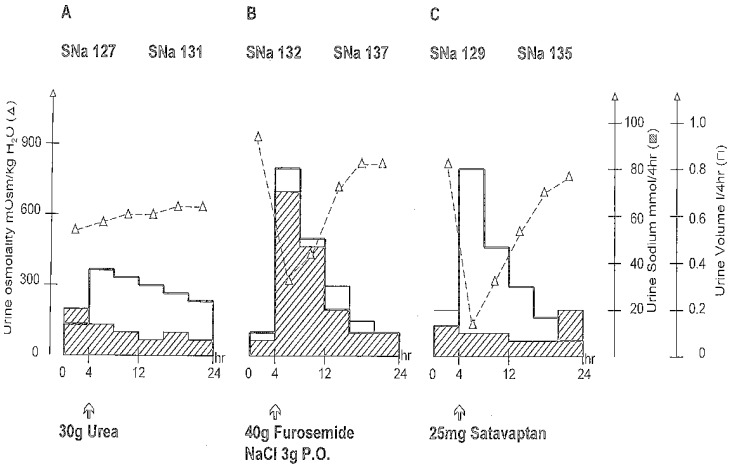
Evolution of diuresis, urine osmolality and urine sodium in three patients with urea, furosemide or V_2_ antagonist (satavaptan) (data adapted form References [22,25,37]).

It is well known that urea induces water loss by increasing the daily osmotic charge eliminated in the urine. During the initial phase of the treatment with urea, normalization of the serum sodium is due to the “antinatriuretic” and osmotic property of urea [13]. During the first days of urea therapy (at daily dose equivalent to those used during chronic therapy) concomitant water restriction is necessary to obtain a negative water balance. A body loss of 1 to 3 kg is necessary usually to normalize the serum sodium. Higher doses of urea can be used during the first day (for example 3 × 30 g/day) if water intake can not be restricted. It the daily urinary osmotic excretion is, for example 700 mOsm/kg/H_2_O/day, an additional osmotic charge of 500 mOsM provided by the administration of 30 g of urea will result in a diuresis of 1500 mL for a fixed urine osmolality of 800 mOsm/kg/H_2_O. If water intake is limited from 1500 to 2000 mL/day, 30 g of urea, either in one or in two divided doses (2 × 15 g), is generally sufficient to maintain a normonatremia. One can calculate, with the aid of the measured urine osmolality, the daily dose of urea required to obtain the additional volume of urine necessary to maintain normonatremia, e.g., 30 g urea (500 mOsm) allows the excretion of 1 L free water if urine osmolality is fixed at 500 mOsm/kg/H_2_O. Oral crystalline urea is dissolved in 100 mL water and is taken after meals to avoid gastric intolerance. The poor palatal taste of the preparation could be avoided by addition of fruit syrup. For patients who do not tolerate oral urea (~15% of patients) we usually prescribed an effervescent solution, used in the past to treat gastric ulcer [2,3] (urea 10 g, NaHCO_3_ 2 g, citric acid 1.5 g and sucrose 200 mg to be dissolved in 50 mL to 100 mL water). Urea can been used for years without toxicity and is an alternative to vasopressin V2 antagonist ([27,28], see Figure 2). In animals, it has also been shown that treatment of hyponatremia with urea has a protective effect against osmotic demyelinating syndrome (ODS) [28,29,30,31,32,33]. Despite the protective effect of urea, we advise to correct hyponatremia with a daily increase of less than 10 mmol/day [26]. For the patients who are overtreated or present early symptoms of ODS we advise to rapidly decrease SNa so that the daily increase in SNa stays lower than 10 mmol/L [26,34,35,36]. Prospective studies showing the benefit to treat mild chronic hyponatremia due to SIADH and comparing water restriction, V2 antagonist, high ceiling diuretics, and urea should be done [26,37].

## 4. Conclusions

These data show that urea is a simple and inexpensive therapy to treat euvolemic hyponatremia.
